# Commentary: Using Directional Deep Brain Stimulation to Co-activate the Subthalamic Nucleus and Zona Incerta for Overlapping Essential Tremor/Parkinson's Disease Symptoms

**DOI:** 10.3389/fneur.2019.00854

**Published:** 2019-09-06

**Authors:** Chengyuan Wu, Caio Matias

**Affiliations:** ^1^Division of Epilepsy and Neuromodulation Neurosurgery, Department of Neurosurgery, Vickie and Jack Farber Institute for Neuroscience, Thomas Jefferson University, Philadelphia, PA, United States; ^2^Department of Radiology, Jefferson Integrated Magnetic Resonance Imaging Center, Thomas Jefferson University, Philadelphia, PA, United States

**Keywords:** deep brain stimulation, directional leads, stimulation side effects, zona incerta, Parkinson's disease, essential tremor, volume of tissue activation, anatomy

We have read with interest the article titled “Using Directional Deep Brain Stimulation to Co-activate the Subthalamic Nucleus and Zona Incerta for Overlapping Essential Tremor/Parkinson's Disease Symptoms” by Falconer et al. (1). In this case report, the authors present a patient treated with unilateral left-sided deep brain stimulation (DBS) after medical management alone proved to be ineffective. The stereotactic target was described as being “3 mm lateral to the most lateral point of the red nucleus” at the Bejjani line (2); and implantation of the electrode at this target was confirmed with microelectrode recordings, intraoperative macrostimulation testing, as well as post-operative imaging. Based on their clinical findings with significant UPDRS improvement and medication reduction, the authors ultimately concluded that DBS in this patient resulted in “possible co-activation of the dorsal aspect of the STN and the adjacent ZI through the utilization of a bipolar directional montage on a single segmented contact.” Although an interesting and exciting concept, we found this interpretation to be misleading.

First of all, the authors have clearly demonstrated the implanted electrode location based on what appears to be a post-operative CT scan merged with a preoperative MRI scan [Figure 3 from Falconer et al. (1)]. There are concerns regarding the accuracy of this image, as registration errors between different imaging modalities can result in misrepresentation of electrode position (3). While we would have preferred to see a post-operative MRI with the electrode in place, we do understand that FDA approval of such imaging was not in place at the time of publication for this case report. Furthermore, the ability of intraoperative CT to accurately represent lead location has been previously reported (4, 5). As such, we shall assume that the registration between CT scan and MRI was accurate and that the final electrode position is properly represented.

In this figure provided by the authors, the electrode clearly straddles the lateral border of the subthalamic nucleus (STN) and abuts the internal capsule (IC). To help further clarify the position of this electrode in three-dimensional space, we have transposed its location onto a set if images adapted from the Schaltenbrand and Warren atlas (Figure 1). The caudal ZI lies medial, posterior, and superior to the STN, ~5 mm from the electrode.

**Figure 1:**
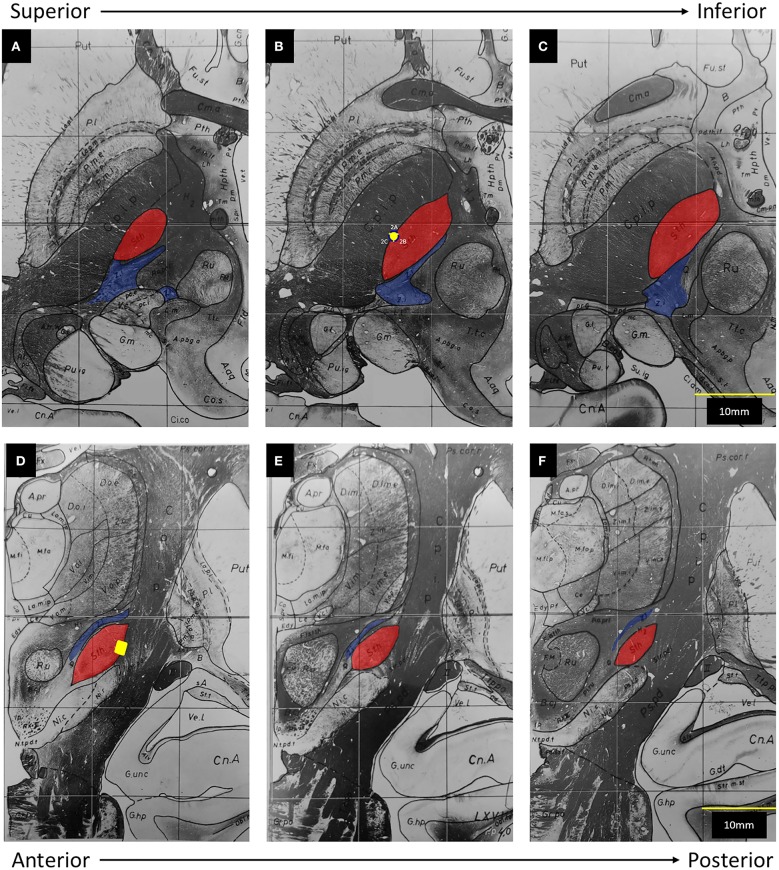
Adapted from Schaltenbrand and Warren (6): The subthalamic nucleus (Sth) has been highlighted in red and the zona incerta (Z.i.) has been highlighted in blue. The posterior limb of the internal capsule (C.p.i.p) lies lateral to the Sth. The yellow dot represents the position of Contact 2 on axial **(A–C)** and coronal **(D–F)** views. **(A)** 1.5 mm below midcomissural point (MCP). **(B)** 3.5 mm below MCP. **(C)** 4.5 mm below MCP. **(D)** 3 mm posterior to MCP. **(E)** 4 mm posterior to MCP. **(F)** 5 mm, posterior to MCP.

In both intraoperative testing and post-operative programming, the authors found that contacts 2B and 3B provided greater therapeutic benefit. Although contact 2B provided the “largest therapeutic window,” a lack of control of the patient's kinetic tremor with monopolar stimulation led to the use of a bipolar configuration.

We have found it interesting that in the entire description of the case, there is a complete lack of reporting of side effects from stimulation—particularly given the location of the implanted electrode. One would expect that use of contacts 2 or 3 in an omnidirectional manner would activate IC fibers, resulting in tonic contractions, facial pulling, or dysarthria. It would make sense that the more medial contacts (2B or 3B) would have to be activated in an isolated fashion in order to avoid such side effects from IC activation. Given their reference to a “therapeutic window,” one would assume that side effects were indeed encountered. Unfortunately, the authors did not report any details of such clinical findings or with regards to activation of contacts 2 or 3 in an omnidirectional manner.

Finally, the authors conclude that activation of segment 2B in a bipolar fashion allowed the current to penetrate through the width of the STN, eventually co-activating zona incerta (ZI) fibers. From our understanding of basal ganglia anatomy and the concept of volume of tissue activated (VTA), the reported parameters of 1.4 mA, 160 Hz, and 60 μs are unlikely to activate ZI. When considering the average red nucleus diameter of 6 mm (7) and the average STN length of 8–9 mm (8, 9), ZI appears to be ~5 mm from the electrode in the figure provided by the authors, which is concordant with atlas measurements (Figure 1). Even when considering the three-dimensional anatomy and the possibility of current spread superiorly, the active contact still resides at least 4 mm away from the rostral ZI. Based on the VTA model for omnidirectional DBS proposed by Mädler and Coenen (10), an amplitude of 1.4 mA would activate fibers within ~2.5 mm. More germane to this case, Buhlmann et al. presented finite element models for both monopolar and bipolar stimulation (11). According to their models, monopolar activation of a single segment did not increase the penetration of the current; and use of bipolar stimulation at twice the amplitude increased the distance of tissue penetration by ~50%. As such, without any increase in amplitude, it is simply not possible to increase the distance of tissue penetration from 2.5 to 4 mm [Supplementary Figure (12)]. In fact, it is more likely that an omnidirectional stimulation from an optimally-placed electrode within the substance of the STN will result in co-activation of the caudal ZI.

Ultimately, this case report demonstrates the ability of directional stimulation to minimize side effects from a laterally positioned electrode, which would be an expected result associated with internal capsule stimulation. The reduction in bradykinesia, rigidity, as well as rest and kinetic tremor on this patient is most likely due to activation of motor STN, which has also been reported to control both rest and intention tremors (13). While monopolar stimulation did not activate a sufficient volume of the superior-posterior-lateral STN, use of bipolar stimulation may have recruited differently oriented white matter fibers (14). Specifically, anodic stimulation may preferentially activate the hyperdirect pathway fibers, while avoiding activation of adjacent internal capsule fibers passing the electrode (15). Certainly, further investigation into the specific effects of bipolar stimulation on directional DBS electrodes is needed.

We must also emphasize, however, that previous studies have shown that long-term outcome correlates with electrode position (16–20). As the patient's Parkinson's disease progresses, the STN will atrophy (21). As this progression leads to increased current requirements (22), a sub-optimally placed electrode may induce stimulation side-effects before therapeutic effect is achieved. While directional DBS electrodes increase programming options, there remains no substitute for optimal electrode placement.

## Author Contributions

CW initiated the need for this general commentary. Both CW and CM wrote sections of the manuscript and created the included figure. Both authors contributed to manuscript revision, read, and approved the submitted version.

### Conflict of Interest Statement

The authors declare that the research was conducted in the absence of any commercial or financial relationships that could be construed as a potential conflict of interest.
