# Supplementary figures and images for: Commentary: Using Directional Deep Brain Stimulation to Co-activate the Subthalamic Nucleus and Zona Incerta for Overlapping Essential Tremor/Parkinson's Disease Symptoms
Source: Front Neurol. 2019 Sep 6;10:854. doi: 10.3389/fneur.2019.00854 (PMC6742774; doi:10.3389/fneur.2019.00854)

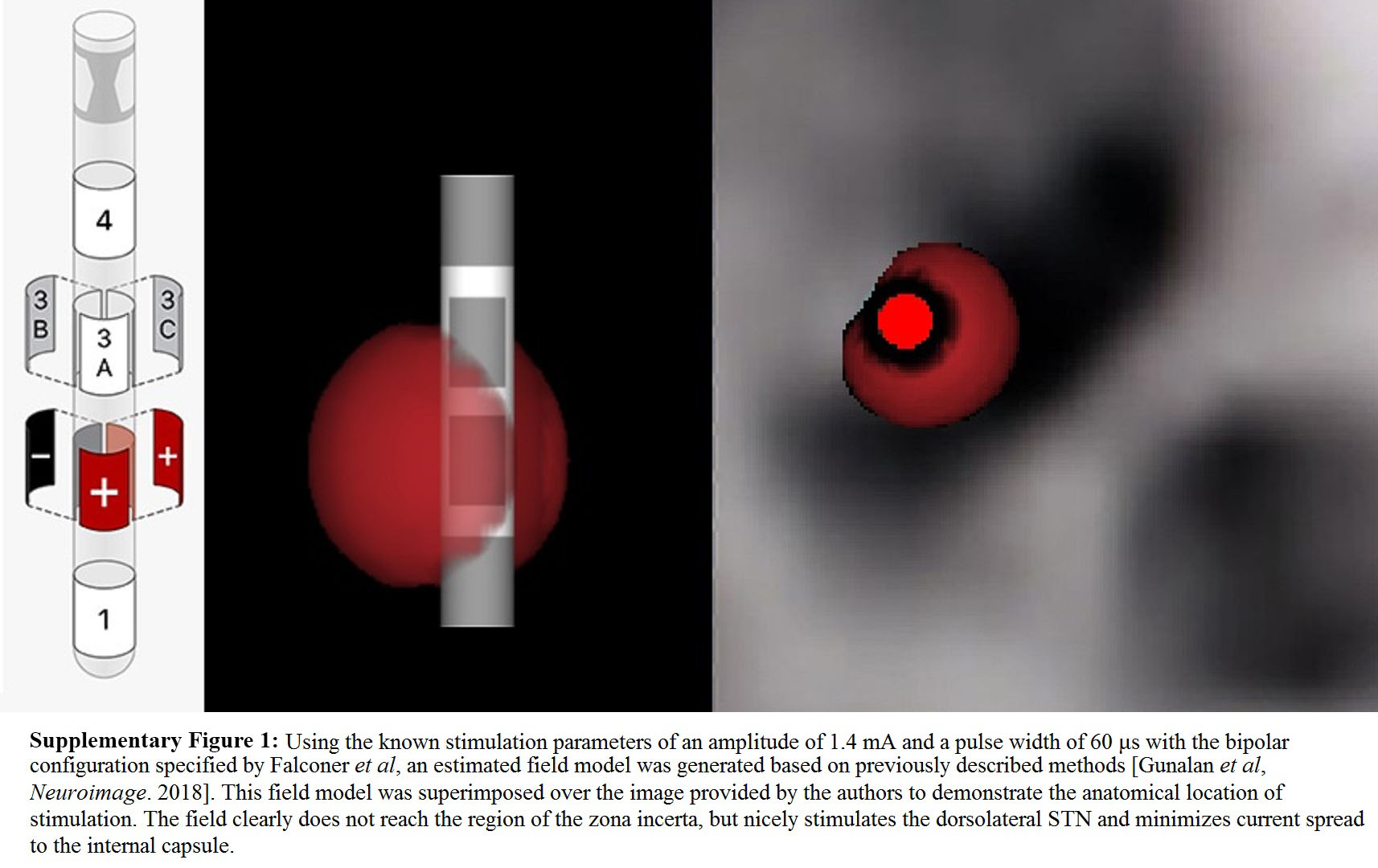

Supplement: Supplementary file 1 [file Image_1.jpg]
